# The Effects of Biomaterial Implant Wear Debris on Osteoblasts

**DOI:** 10.3389/fcell.2020.00352

**Published:** 2020-06-03

**Authors:** Li Zhang, El-Mustapha Haddouti, Kristian Welle, Christof Burger, Dieter C. Wirtz, Frank A. Schildberg, Koroush Kabir

**Affiliations:** Clinic for Orthopedics and Trauma Surgery, University Hospital Bonn, Bonn, Germany

**Keywords:** total joint arthroplasty, wear particles, aseptic loosening, osteoblasts, osteoclasts, macrophages

## Abstract

Aseptic loosening subsequent to periprosthetic osteolysis is the leading cause for the revision of arthroplasty failure. The biological response of macrophages to wear debris has been well established, however, the equilibrium of bone remodeling is not only dictated by osteoclastic bone resorption but also by osteoblast-mediated bone formation. Increasing evidence shows that wear debris significantly impair osteoblastic physiology and subsequent bone formation. In the present review, we update the current state of knowledge regarding the effect of biomaterial implant wear debris on osteoblasts. The interaction of osteoblasts with osteoclasts and macrophages under wear debris challenge, and potential treatment options targeting osteoblasts are also presented.

## Introduction

Total joint arthroplasty (TJA) has been widely and successfully applied for surgical management of severe trauma or arthritic joint diseases. It restores joint function, alleviates pain, and enhances the quality of life. However, the long-term implant survival rate decreases with time, potentially leading to implant failure, which requires revision surgery (Marshall et al., 2008). Causes of implant failure are multifactorial, and include material properties, component design, surgical techniques, biomechanical factors, host response, periprosthetic fracture, infection, and implant loosening. Aseptic loosening is the leading cause for revision surgery, accounting for over 75% of all cases (Hallab and Jacobs, 2009) and plays a predominant role in limiting the longevity of current TJAs. As the only established treatment for periprosthetic osteolysis to date, revision surgery is technically complex, and is associated with a high rate of complications, high morbidity, poor clinical and functional performance, as well as having a significant economic impact on the healthcare system (Bozic et al., 2015). In this regard, an understanding of the specific mechanisms underlying failure of primary TJA is essential to reduce the necessity for TJA revision and to improve implant longevity.

Wear resistance of implants and the biologic reactivity of wear particles play a key role in long-term implant survival. Particulate debris are generated at the implant surface because of wearing, which then migrate to and infiltrate surrounding tissue. The size, morphology (Yang et al., 2002), volume, and composition (Lohmann et al., 2000) of wear particles are associated with their biologic reactivity, and are important determinants of peri-implant cell fate (Stratton-Powell et al., 2016). Phagocytosed particles ranging in size between 0.1 μm and 10 μm are considered the most biologically reactive. Particles with smaller sizes, particularly those with a mean size of <1 μm, are likely to induce a stronger inflammatory response than larger ones (Vermes et al., 2000). The effects of implant wear debris during osteolysis have been studied using particles from metallic implants [titanium (Ti), cobalt (Co), chromium (Cr)], polymeric implants [polymethylmethacrylate (PMMA)] and ceramic implants [alumina (Al_2_O_3_), zirconia (ZrO_2_)] (Gibon et al., 2017a, b; Klinder et al., 2018). Scanning electron microscopy has revealed that particulate materials generated from implant wear vary more greatly in shape than commercially available particles (mostly spherical), and are more detrimental or inflammatory to osteoblasts (Dean et al., 1999b). However, the effect of the specific morphology of wear debris on their bioreactivity has, do date, not been fully investigated (Stratton-Powell et al., 2016). Other wear products, such as short chain alkane polymers (Maitra et al., 2009) and metal ions (Magone et al., 2015; Jonitz-Heincke et al., 2017), can also trigger an additional toxicity in cells; however, this will not be discussed here.

Although it is known that the chronic inflammatory reaction that occurs in response to biomaterial wear debris is mostly driven by macrophages, osteolysis is also indirectly caused by a partial contribution of several cell types such as osteoblasts, dendritic cells, osteoclasts, and synovial fibroblasts (Limmer and Wirtz, 2017; Li et al., 2018). Upon wear particle challenge, macrophages release an array of proinflammatory mediators, which trigger the recruitment, multiplication, differentiation, and maturation of osteoclast precursors. Subsequent bone resorption ultimately results in prosthetic loosening. However, there is growing evidence that, except for activating bone resorption, wear debris also impair new bone generation by inhibiting osteoblast function. Moreover, the role of osteoblasts in the development of periprosthetic bone loss has, to date, not been fully explored. Thus, elucidation of the response of osteoblasts to wear debris is critical to obtain an understanding of the problem of implant loosening as a whole.

In the present review, we briefly introduce the general cellular response of osteoblasts to various biomaterial wear particles and discuss the interactions between osteoblasts and macrophages/osteoclasts. In particular, the emerging strategies for aseptic osteolysis targeting osteoblasts are summarized by listing representative signaling pathways and pharmacological candidates from *in vitro* and *in vivo* models.

## Osteoblasts

Osteoblasts, which comprise a diverse population of cells that develop from pluripotent mesenchymal stem cells, include immature preosteoblasts as well as differentiating and mature matrix-producing osteoblasts. As the chief bone-forming cells, they are involved in the production of extracellular matrix constituents including osteocalcin, alkaline phosphatase, and a large amount of type I collagen, which constitutes 90% of the organic bone matrix. Osteoblasts also regulate extracellular matrix mineralization by secreting matrix vesicles (Yang et al., 2002; Boonrungsiman et al., 2012). Further, osteoblasts regulate osteoclasts by secreting the receptor activator of nuclear factor (NF)-κB ligand (RANKL) and osteoprotegerin (OPG). Osteoblast differentiation is controlled by RUNX2 (runt-related transcription factor 2), WNT, and BMP signaling pathways. Following matrix formation, osteoblasts have three possible fates: (1) they differentiate into osteocytes entombed within the mineralized bone matrix, (2) they undergo apoptosis, or (3) they transform into inactive quiescent bone-lining cells (Bonewald, 2011).

Osteoblasts are responsible for bone formation but can indirectly participate in bone degeneration by changing cell viability and expression of specific chemokines as well as directly through the secretion of preosteolytic mediators and specific proteinases. Of particular importance in the context of this review article is the ability of osteoblasts to internalize wear particles, to demonstrate cellular dysfunction, and to contribute to wear particle-induced osteolysis. During osteolysis, although they may not be the major player as macrophages, osteoblasts still play a crucial synergetic role by coordinating with macrophages and osteoclasts. To date, diverse *in vitro* cell models have been used to explore osteoblasts’ reactions to wear particles; these include models using osteosarcoma cell lines (MG-63, SaOS-2 and U-2 OS), mouse stromal precursor cells (MC3T3-E1), and primary human osteoblasts (hOBs). When summarizing the *in vitro* studies in this review, we mainly focus on these four cell lines.

## Biological Response of Osteoblasts to Orthopedic Wear Debris

### Interaction Between Osteoblasts and Wear Debris

Osteoblasts interact with particles *via* phagocytic and non-phagocytic mechanisms. Recently, Toll-like receptor (TLR) signaling has been implicated in the response of macrophages to wear particles and the subsequent inflammatory reaction (Jämsen et al., 2017). However, whether TLRs are involved in osteoblast-particle interaction has not been fully clarified (Jonitz-Heincke et al., 2019). Although they are thought to be non-phagocytic cells, osteoblasts have been shown to engulf and internalize particulate debris within the osteoblast cytoplasm (Vermes et al., 2001; Chiu et al., 2010). Pretreatment with cytochalasin D, a potent phagocytosis inhibitor that disrupts the assembly of actin filament, significantly suppressed particle internalization (Vermes et al., 2000, 2001; Lee et al., 2011) and mitigated particle-mediated functional changes such as viability, differentiation, and inflammatory reaction (Pioletti et al., 1999; Shida et al., 2000; Vermes et al., 2000; Fritz et al., 2006; Morishige et al., 2010; Burton et al., 2013), implying the requirement of phagocytosis for particle-osteoblast interaction. Osteoblasts also internalize wear debris through other pathways. Indeed, osteoblasts have been reported to internalize particles *via* micropinocytosis-, clathrin- and caveolin-mediated endocytosis (Shi et al., 2018; Jin et al., 2018). However, so far, very few studies have been reported on this topic. Particles can also interact with cells in a non-phagocytozable manner during contact, although probably to a lesser degree (Vermes et al., 2000; Granchi et al., 2004). Interestingly, particles of phagocytozable size that are not internalized through the phagocytosis pathway can also interact with osteoblasts, because the inhibitory effect of cytochalasin D on the cellular response is incomplete (Pioletti et al., 1999; Vermes et al., 2001; Fritz et al., 2006).

As wear particle internalization is important for a cellular reaction to particles (e.g., cytotoxicity, inflammatory reaction), the internalization pathway could also be a potential target for particle-induced osteolysis. However, to date, the individual pathways for different wear particle internalization in peri-implant cells have not been identified, thus, more studies are required on this topic.

### Intracellular Organelle Damage

The cytoplasm of osteoblasts is rich in organelles such as well-developed rough endoplasmic reticulum, large Golgi complex, transfer vesicles, secretory granules, and electron-dense mitochondria that are necessary for the secretion of extracellular matrix proteins. After incorporation of wear debris, osteoblasts exhibit ultrastructural changes in actin fibers, cell membranes, mitochondria, endoplasmic reticulum, and Golgi bodies (Lohmann et al., 2000, 2002a; Lee H. G. et al., 2012; Ribeiro et al., 2016). Actin staining revealed significantly pronounced particle attachment on actin fibers and disorganized actin filaments (Kwon et al., 2000; Saldaña and Vilaboa, 2010; Lee et al., 2011; Lee H. G. et al., 2012). In addition, osteoblasts developed swelling of intracellular organelles associated with cell-membrane disruption, exhibited large Golgi extensions, reduced numbers of rough endoplasmic reticulum, and enlarged, swollen mitochondria and lysosomal-like elements. Further, although no particles were found in the nucleus, supranuclear vacuolization, cell cycle arrest in G2/M phase, and an increased quantity of DNA fragments were observed, suggesting DNA damage and DNA repair activation (Ribeiro et al., 2016). The presence of intracellular lysosomal-like elements might serve as evidence of the direct cytotoxic effect of particles on the cells.

### Cell Cytoskeleton Disruption

Osteoblasts also exhibit changes in actin fibers. Cytoskeleton staining revealed significantly disrupted cytoskeletal architecture, supported by pronounced particle attachment on actin fibers and disorganized actin filaments (Kwon et al., 2000; Saldaña and Vilaboa, 2010; Lee et al., 2011; Lee H. G. et al., 2012). It has been noted that the cell cytoskeleton is linked to cellular elasticity and sensing of mechanical changes. Even wear particles that exert no cytotoxicity, may therefore, still be potentially deleterious to the long-term cell physiology due to changes of a subsequent event cascade to cytoskeletal organization. However, although changes in cytoskeletal rearrangement have been reported in wear particle-challenged osteoblasts, few studies have investigated the change in cytoskeleton-dependent cell function, especially osteoblastic mechanosensing and cell differentiation. Collectively, cytoskeletal disruption might hamper cellular functions, although the exact mechanisms and pathways remain to be analyzed.

### Alteration in Viability, Proliferation, Adhesion, and Migration

Several studies have extensively demonstrated general detrimental effects of different kinds of wear particles on a number of osteoblastic functions including viability (Choi et al., 2005), proliferation (Vermes et al., 2001; Saldaña and Vilaboa, 2010; Yang et al., 2015), adhesion (Kwon et al., 2000; Choi et al., 2005; Saldaña and Vilaboa, 2010; Hou et al., 2013; Preedy et al., 2015), and migration (Saldaña and Vilaboa, 2010) in a particle composition-, size-, dose-, and time-dependent manner. In studies where particles have been found to exhibit no harmful effects on viability or proliferation, the concentrations of these particles (for example: 0.1 mg/mL for Ti and 0.5 mg/mL for UHMWPE) were selected as sub-cytotoxic doses for future work.

Various studies have shown that these responses vary with particle material composition. Particulate debris of alumina and polystyrene origins are less harmful than those originating from metals and polymers. According to Dalal et al. (2012) MG-63 cells demonstrated greater cytotoxicity in response to CoCrMo particles than to other metal particles (Ti, Zr-Oxide, Zr alloy) after 48 h, with a concentration of 1 μm of CoCrMo particles eliciting the strongest suppression of viability and proliferation. In line with these findings, Lenz et al. (2009) have reported that CoCrMo particles caused more severe suppression of viability, while Ti and ZrO_2_ showed no effects. Notably, particulate CoCr appeared to be less harmful than either of its major metal constituents (Allen et al., 1997; Rose et al., 2012). Particle exposure also hampers osteoblastic cell adhesion and migration. Cell cytoskeleton and assembly of actin filaments enables the maintenance of a stable cellular shape as well as cell adhesion and motility. Disassembly of cytoskeletal structures due to particle exposure is expected to account for the loss of a crucial number of associated cellular functions. Various particles have been demonstrated to impair the spreading and adhesion strength in osteoblasts (Kwon et al., 2000, 2001; Saldaña and Vilaboa, 2010; Drynda et al., 2018).

### Osteogenic Differentiation

Osteogenic differentiation and mineralization of osteoblasts are also impaired on exposure to wear particles. These effects are evidenced by the reduced gene expression of early bone-formation-associated genes [e.g., *ALP, runx 2, osterix* (Dean et al., 1999a; Lohmann et al., 2000; Shen et al., 2014; Gu et al., 2017)] and late osteogenic markers (e.g., osteocalcin) (Nam et al., 2017), whilst simultaneously losing their capacity to synthesize type I collagen (Allen et al., 1997; Yao et al., 1997; Vermes et al., 2000, 2001; Lochner et al., 2011; Schulze et al., 2013) and mediate mineralization (Gu et al., 2017). Additionally, similar trends were observed in the expression of osteoblastogenesis-related transcription factors such as osteopontin (OPN), TGF-β1, sp7, bgalp, Fra-2 (fos related antigen-2), Dlx5, and Dkk1 (dickkopf-related protein 1), even though some of these trends were not statistically significant (Chiu et al., 2010; Ma et al., 2010; Shen et al., 2014; Sun et al., 2015; Deng et al., 2017b; Gu et al., 2017). In parallel, the expression of negative regulators of osteogenesis, such as BMP3, sclerostin (SOST), and Msx2, is found to be temporarily up-regulated during osteogenesis (Chiu et al., 2010; Ma et al., 2010). The particle-induced inhibition of osteogenic differentiation is mainly mediated by Wnt/β-catenin and BMP/smad signaling pathways, as both BMP and WNT signaling were suppressed in osteoprogenitor cells (Sun et al., 2015; Nam et al., 2017; Sun et al., 2019). Thus, these two signaling pathways could be used as a potential therapeutic target. This aspect will be discussed later.

### Extracellular Matrix Imbalance

Through the secretion of extracellular matrix and effectors such as the matrix degradative proteinases MMPs (metalloproteinases) and their inhibitors TIMP (tissue inhibitors of metalloproteinases), osteoblasts actively contribute to peri-implant matrix remodeling. However, pathological periprosthetic matrix remodeling will contribute to periprosthetic loosening. Extracellular matrix (osteoid) mainly consists of type I collagen. Wear particles have been reported to suppress type I collagen synthesis to a varying degree in osteoblasts (Allen et al., 1997; Yao et al., 1997; Vermes et al., 2000, 2001; Lochner et al., 2011; Schulze et al., 2013). This inhibitory effect is related to particle size, dose, and composition. Lochner et al. (2011) reported that at the concentration of 0.1 mg/mL, particles from both Co and Cr significantly inhibited the release of procollagen type I. This suppressive effect is dependent on the tyrosine phosphorylation cascade targeting the NF-κB signaling pathway (Vermes et al., 2000; Roebuck et al., 2001).

Wear debris have been reported to disrupt the balance between osteoblastic MMPs and TIMP which would result in limited implant osseointegration and subsequent implant failure (Ma et al., 2006; Syggelos et al., 2013). Takei et al. (2000) observed highly elevated mRNA expression of 16 different types of MMPs in synovium-like interface tissues from loose artificial hip joints that required revision surgery. This indicates the pro-osteolytic effect of MMPs in implant failure. Jonitz-Heincke et al. (2016) reported that primary hOBs showed significant up-regulation of MMP-1 and MMP-8 and down-regulation of MMP-3 and MMP-10 (Jonitz-Heincke et al., 2016) after *in vitro* exposure to abrasive endoprosthetic wear particles. Additionally, they found that the synthesis of TIMP-1 and TIMP-2 was inhibited. Klinder et al. (2018) found elevated *MMP-1* and *TIMP-1* expression in hOB after CoCr and alumina particles. CoCr induced a more prominent MMP-1 elevation than alumina. Other pro-osteolytic mediators of the MMP family, such as *MMP-2* (Chen et al., 2014) and *MMP-13* (Ormsby et al., 2016) were also observed to be upregulated in osteoblasts. In contrast, *TIMP-2* expression remains unchanged. Notably, Sasaki et al. (2001) identified a significant up-regulation of *TIMP-1*, *-2*, and *-3* mRNA, and decreased *TIMP-4* mRNA, in the periprosthetic tissue of artificial hip joints that had undergone aseptic loosening. This *in vivo* TIMP increase may be a reaction to the increased local production of MMPs; it also suggests that the *in vivo* activity of MMPs may not be effectively inhibited by TIMPs, thus resulting in unrestricted matrix degradation.

### Production of Inflammatory Mediators

During aseptic loosening, inflammatory reactions are mainly driven by activated macrophages, especially M1 macrophages (Nich et al., 2013). Osteoblasts, osteocytes, fibroblasts, and dendritic cells also contribute to peri-implant inflammation. Pro-inflammatory mediators not only promote osteoclastic bone resorption but also restrain new bone formation. This ultimately results in osteolysis. A summary of major pro-inflammatory mediators in retrieved human periprosthetic tissue during revision arthroplasty is presented in Table 1. Wear particles activate osteoblasts to secrete inflammatory mediators (Wang C.-T. et al., 2010; Vallés et al., 2013). Gene expression and secretion of osteoblastic proinflammatory cytokines (TNFα, IL-1β, IL-6, and M-CSF) are significantly up-regulated in a time- and dose-dependent manner (Lochner et al., 2011) after exposure to wear debris. These factors further exacerbate osteoclast activity and ultimately lead to bone loss. Among these, TNFα is the predominant proinflammatory factor and has been shown to control the release of other proinflammatory mediators, such as IL-1β and IL-6, *via* autocrine and paracrine signaling (Wang C.-T. et al., 2010). Dalal et al. (2012) exposed osteoblasts to various metal-based particles (CoCrMo, Ti, Zr-Oxide, Zr alloy) and found that inflammatory mediators (IL-6 and TNFα) were markedly up-regulated in response to all particle types, with CoCrMo particles producing the strongest effects. Interestingly, cytokines such as TNFα and IL-1β can alter collagen matrix formation by osteoblasts, which further underlines their importance in periprosthetic tissues (Hozhabri et al., 2015). Similarly, osteoblasts show increased PGE2 secretion after particle phagocytosis *in vitro* (Dean et al., 1999a; Lohmann et al., 2000, 2002a,b; Dean et al., 2001; Vallés et al., 2008b). This increase also depends on particle concentration (Dean et al., 1999a, 2001; Lohmann et al., 2002b), composition (Lohmann et al., 2000), as well as osteoblastic maturation state (Lohmann et al., 2002a).

**TABLE 1:** Major pro-inflammatory mediators which are relevant to the context of orthopedic implant pathology in retrieved human periprosthetic tissue.

**Pro-inflammatory mediator type**	**Mediators**	**Pathogenic role on wear particle-induced osteolysis**	**References**
Inflammatory cytokines	TNF-α IL-1α IL-1β IL-6 M-CSF	Promote RANKL and osteoclast differentiation, activation, and survival. Stimulate osteoclast activity, disrupt MMP/TIMP balance, promote bone resorption Inhibit osteoblastogenesis, reduce bone formation	Jämsen et al., 2014; Christiansen et al., 2019
Chemokines	CXCL8 (IL-8) CCL2 (MCP-1) MIP-1 Others: CCL4/9/10/22 et al.	Induce both local and systemic cell trafficking of monocytes/macrophages to the bone-implant interface Cell apoptosis, angiogenesis, collagen production, and tissue remodeling	Goodman and Ma, 2010; Jämsen et al., 2014
Other mediators	iNOS	Synthesis of NO, promote osteoclastic bone resorption	Hukkanen et al., 1997; Lavigne et al., 2002
	COX-2	Synthesis of PGE2, promote osteoclastic bone resorption	

*M-CSF, macrophage colony-stimulating factor; RANKL, receptor activator of nuclear factor kappa-*B* ligand; MMP, metalloproteinases; TIMP, tissue inhibitors of metalloproteinases; CXCL-8, C-X-C Motif Chemokine Ligand 8; CCL2, C-C Motif Chemokine Ligand 2; MCP-1, monocyte chemoattractant protein 1; iNOS, inducible nitric oxide synthase; NO, nitric oxide; COX-2, cyclooxygenase-2; PGE2, prostaglandin E2.*

Although macrophages and MSCs have been implicated as the major source of chemotactic cytokines (chemokines) in periprosthetic tissues secreted in response to different types of wear particles, osteoblasts are also involved in the production of chemokines such as monocyte chemoattractant protein-1 (MCP-1, also known as CCL2) and IL-8 (or CXCL8). It has been demonstrated that *IL-8* and *MCP-1* mRNA expression and protein secretion were up-regulated when osteoblasts were exposed to implant particles, in a time- and concentration-dependent manner *in vitro* (Fritz et al., 2002, 2006; Queally et al., 2009; Lochner et al., 2011; Yang et al., 2015), and that this up-regulation was dependent on particle-induced NF-κB-mediated transcriptional activation. These chemokines play critical roles in the initiation of inflammation processes by local and systemic recruitment of inflammatory cells (e.g., monocytes, T cells, and neutrophils) to the site of particle generation (Goodman and Ma, 2010; Dyskova et al., 2017; Nabeshima et al., 2017).

### Imbalance of OPG/RANK/RANKL

Particle-challenged osteoblasts may contribute to periprosthetic osteolysis through a RANKL-dependent pathway (Jiang et al., 2013). RANKL binds to RANK, triggering the cascade of intracellular signaling pathways (NF-κB) that are essential for osteoclast differentiation and activation (Cordova et al., 2015; Kim et al., 2015). Osteoprotegerin (OPG), a known osteoclastogenesis-inhibitory factor, acts as a “decoy receptor” for RANKL secreted by osteoblasts. Previous *in vitro* studies have demonstrated that significantly elevated RANKL gene expression and OPG suppression resulted in a much higher RANKL/OPG ratio than in controls, when osteoblasts were challenged with particulate Co-Cr alloy or UHMWPE (Granchi et al., 2004, 2005; Vallés et al., 2008b; Yang et al., 2015). Furthermore (Granchi et al., 2004, 2005) reported that a large number of multinucleated TRAP-positive giant cells was obtained when peripheral blood mononuclear cells (PBMCs) were co-cultured in conditioned medium from hOBs after UHMWPE challenge, and that these cells showed significantly increased RANKL/OPG expression. In contrast, Al_2_O_3_ wear debris elicited weaker reactions than UHMWPE (Granchi et al., 2004, 2005). Moreover, an *in vivo* study reported that locally delivered *RANKL* siRNA inhibited osteolysis and increased bone formation in an UHMWPE particle-induced osteolysis model (Cordova et al., 2015). These data suggest that the RANKL/OPG pathway is involved in wear particle-promoting osteoclastogenesis and could therefore represent a potential therapeutic target.

### Autophagy

Autophagy is an evolutionarily conserved process that plays important roles in cellular homeostasis both under normal conditions or in response to stressors (Hocking et al., 2012). Numerous studies suggest that autophagy modulates osteoblastic function (Kaluđerović et al., 2014; Nollet et al., 2014; Wang et al., 2015b; Kim et al., 2016; Kang et al., 2017; Tang et al., 2018). Also, autophagy has been reported to be triggered by wear particles in osteoblasts (Wang et al., 2015b). It may therefore be a key factor in the osteoblastic response to wear particles.

Notably, the role of autophagy and wear particle-triggered cytotoxicity remains confusing. Autophagy promotes cell survival in response to wear particles (Kang et al., 2017), however, it can also promote cell death and inflammation (Liu et al., 2016; Xian et al., 2020). The dual role could be attributed to the fact that autophagy plays protective roles within its threshold range, which may be attributed to their role in anti-oxidative stress (Zhang et al., 2016), clearance of material and damaged cellular organelles. However, when particle concentration continues to increase, the protective effect may reach its limit, and therefore cell death is inevitable (Wang et al., 2015b; Yang et al., 2016). Consistent with this notion, a discrepancy between autophagy and osteoblastic cell survival under wear particle challenge has been observed. Ta nanoparticles induced autophagy in MC3T3-E1 cells and promoted cell viability at a low concentration. This was indicated by upregulated LC3-II protein expression, autophagic vesicle ultrastructures, and downregulated p62 expression, suggesting an active cytoprotective role through degradation of hazardous substances. However, suppressed viability was observed at concentrations ≥25 μg/mL as autophagosome degradation was inhibited and autophagic flux was impaired (Kang et al., 2017; Wang et al., 2020), as the degradation of p62 was not continuously increased. The effect was further confirmed using the autophagy inducer rapamycin and the autophagy inhibitor 3-methyladenine (3-MA). Similarly, Wang et al. (2015b) demonstrated that CoCrMo metal particles could induce autophagy-mediated MC3T3-E1 osteoblast apoptosis, both *in vitro* and *in vivo*, and promote osteolysis in an animal model of particle-induced osteolysis (PIO) (Wang et al., 2015b). This suggests that autophagy inhibition mitigated osteolysis in animal models. This is consistent with the result from macrophages (Liu et al., 2016; Xian et al., 2020) and osteoclasts (Wang et al., 2017). Interestingly, nano-sized alumina particles could evoke autophagy process and counter Ti particle-induced apoptosis, NF-κB activation and inflammatory reactions both in MG-63 cells and a mouse calvarial osteolysis model (Zhang et al., 2020). Activation of the tumor necrosis factor ligand superfamily member 12 (TWEAK)-p38 pathway by hyperoside can decrease autophagy and increase cell viability and proliferation, and thus protect MC3T3-E1 cells against Ti particle-induced damage (Zhang and Zhang, 2019).

The above evidence suggests that targeting autophagy may be a potential therapeutic approach for treating particle-induced peri-implant osteolysis. However, the specific threshold range of particles (from different types of materials) to induce autophagy and the exact role of autophagy still remains unclear.

In summary, wear particles affect osteoblasts by disrupting their cellular function (Table 2). These effects may alter the balance between osteoblastic bone formation and osteoclastic bone resorption, thereby promoting periprosthetic osteolysis.

**TABLE 2:** Most frequently used particles and their overall effect on osteoblasts.

**Biomaterial**	**Particle type**	**Particle size**	**Osteoblastic reaction**	**References**
Metal particles	Titanium-based (pure-Titanium, TiO_2_, Ti-6Al-4V et al.) Cobalt-chromium-molybdenum Tantalum	Nanometer to Micrometer	1. Particle internalization 2. Intracellular organelle damage 3. Cell cytoskeleton disruption 4. Cell viability, proliferation, adhesion, migration change 5. Osteogenic differentiation inhibition 6. Extracellular matrix imbalance 7. Inflammatory mediators production 8. OPG/RANKL imbalance 9. Autophagy induction/inhibition	Jin et al., 2018 Ribeiro et al., 2016 Lee H. G. et al., 2012 Preedy et al., 2015 Yang et al., 2015 Gu et al., 2017 Nam et al., 2017 Sun et al., 2019 Chen et al., 2014 Lochner et al., 2011 Nabeshima et al., 2017 Yang et al., 2015 Wang et al., 2020
Polymeric particles	Polyethylene (UHMWPE et al.) PMMA			
Ceramic particles	Al_2_O_3_ ZrO_2_			

*TiO_2_, titanium dioxide; UHMWPE, ultra-high molecular weight polyethylene; PMMA, polymethyl-methacrylate; Al_2_O_3_, aluminium oxide; ZrO_2_, zirconium dioxide; OPG, osteoprotegerin; RANKL, receptor activator of nuclear factor kappa-*B* ligand.*

## Biological Response of Osteocytes and Bone-Lining Cells to Orthopedic Wear Debris

Osteocytes are the longest-lived bone cells, accounting for 90–95% of all cells in mineralized bone. In contrast, osteoclasts and osteoblasts make up approximately 5% of bone tissue cells (Shiflett et al., 2019). Osteocytes embedded in lacuna within the mineralized matrix form dendritic processes within canaliculi to communicate with neighboring osteocytes and other cell lineages (Zhang et al., 2012). *Via* this syncytial network, osteocytes can sense the local and systemic environment within the bone.

Osteocytes have been shown to respond to implant wear particles of multiple orthopedic material types. Under wear particle stimulation, osteocytes directly contribute to a special type of bone loss, known as perilacunar remodeling, through osteocytic osteolysis. According to Ormsby et al. (2016) osteocytes exposed to UHMWPE particles showed increased expression of catabolic markers such as cathepsin K and tartrate-resistant acid phosphatase (TRAP) *in vitro*. Consistent with these findings, histological analysis of calvarial sections from mice and biopsies from total hip arthroplasty (THA) patients with periprosthetic osteolysis showed significantly increased osteocyte lacunar size, suggesting that UHMWPE particles directly induce the loss of osteocytic perilacunar bone (Ormsby et al., 2016). Recently, the same group suggested that osteocytic bone resorption may be specific to females, suggesting the involvement of a gender-specific mechanism in wear particle-induced bone loss (Ormsby et al., 2020).

Osteocytes also regulate bone resorption indirectly in a pro-osteoclastic manner. Similar to osteoblasts, mature osteoblastic cells switch from an anabolic to a more catabolic phenotype under wear particle exposure (Atkins et al., 2009). Cells exposed to different particles showed apoptosis and increased mRNA expression of inflammatory cytokines (Lohmann et al., 2002a; Kanaji et al., 2009; Zhang et al., 2012) as well as osteocytic markers E11, DMP1, and SOST (Zhang et al., 2012) *in vitro*. Additionally, increased expression of genes promoting osteoclast formation and activity (*RANKL*, *M-CSF*, and *IL-8*), and decreased expression of *OPG* mRNA (Atkins et al., 2009) and IFN-β was observed (Wang et al., 2017). These effects may exacerbate osteoclastic bone resorption.

Recent studies have reported that quiescent bone-lining cells represent an alternative to MSCs as a source of osteoblasts (Matic et al., 2016). However, interactions between wear debris particles and bone-lining cells are yet to be explored. This could be attributed to the difficulties in accessing bone lining cells and the lack of markers for identifying and tracing them.

## Fibroblasts and Wear Particles

Fibroblasts constitute 70% of the cells in the pseudosynovial membrane. However, in studies on the pathology of wear-debris-associated osteolysis, they have received far less attention than macrophages, which constitute only 15% of the cells in this membrane. Several studies have demonstrated that fibroblasts contribute to wear particle-induced osteolysis. In addition to *in vitro* studies focusing on fibroblast cytotoxicity and inflammation (Germain et al., 2003; Rose et al., 2012), wear particles have been shown to induce fibroblastic RANKL expression through PGE2 receptor EP4 signaling (Tsutsumi et al., 2009), TLR-MyD88-RANKL (Wang H. et al., 2018), and the ER stress pathway (Wang et al., 2015a, c) both *in vitro* and *in vivo*. Interestingly, autophagy seems to play a complex role in the interaction between wear particles and fibroblasts. Wang et al. (2015b) reported that nano-sized Al_2_O_3_ wear particles promote fibroblastic autophagy, which negatively regulated RANKL expression and osteolysis, both *in vitro* and *in vivo* (Wang C. et al., 2018). In contrast, impaired autophagy in fibroblasts promoted monocyte recruitment by increasing the release of CX3CL1 (C-X3-C motif chemokine ligand 1) (Wu et al., 2019).

## The Interactions Between Osteoblasts and Other Key Cells in Aseptic Loosening

**FIGURE 1:**
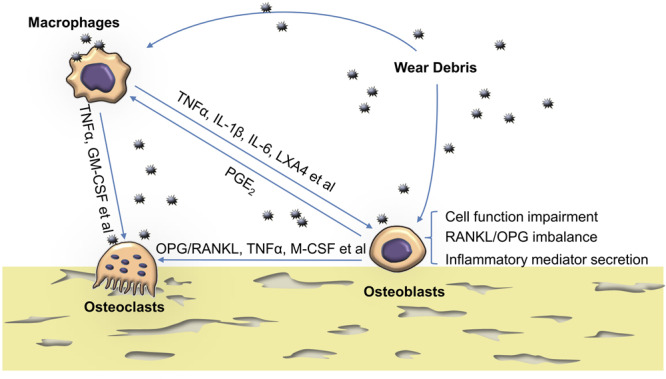
The reaction of osteoblasts to particles and their crosstalk with osteoclasts and macrophages. Wear particles hamper osteoblast cellular function such as viability, proliferation, adhesion, migration, osteogenesis, and matrix mineralization. They also disturb RANKL/OPG balance and increase proinflammatory cytokine production in osteoblasts. Activated macrophages also up-regulate inflammatory cytokines and, together with osteoblasts, contribute to a peri-implant chronic inflammatory environment which favors osteoclastogenesis. TNFα, tumor necrosis factor α; GM-CSF, granulocyte macrophage colony-stimulating factor; PGE2, prostaglandin E2; LXA4, Lipoxin A4; IL, interleukin; OPG, osteoprotegerin; RANKL, receptor activator of nuclear factor kappa-B ligand.

At the peri-implant region, osteoblasts are in close contact with osteoclasts, macrophages, and fibroblasts of the synovial membrane (Horowitz et al., 1994; Lind et al., 1998). Because of this spatial proximity, interaction between these key cell types is inevitable (Okamoto et al., 2017). To explore this in more detail, *in vitro* methods using a conditioned medium have been used. In addition, co-culture systems containing different cell types have been employed as they mimic the *in vivo* environment more closely. Elucidating the interaction of these cell types in the context of wear particle-induced pathology is a crucial step in understanding the problem of implant loosening as a whole (Figure 1).

### Osteoblast-Macrophage Interaction

Osteoimmunological interactions are critical to maintaining bone homeostasis and are key mechanisms in bone pathology. Macrophages can produce many osteo-active factors, such as BMP-2 and transforming growth factor β (TGF-β), to direct osteoblastic activity (Pettit et al., 2008). During the progression of wear particle-induced peri-implant osteolysis, the macrophages are systemically recruited to the local site of particle generation. Notably, osteal tissue macrophages (OsteoMacs) are a resident macrophage population in osteal tissues. They constitute approximately one sixth of the total cells and have been recounted as an important player in bone modeling through directing osteoblast function/mineralization (Cho, 2015). However, their contribution in osteoblast-macrophage interaction during aseptic loosening has never been studied and requires further exploration.

The communication between macrophages and osteoblasts during aseptic loosening has been studied in direct or indirect co-culture models. Upon particle stimulation, co-cultured macrophages secrete soluble factors such as TNFα, IL-6, IL-1β, and GM-CSF (Horowitz and Gonzales, 1996; Rodrigo et al., 2002; Vallés et al., 2008a; Guo et al., 2013) and stimulate the release of osteoblastic IL-6, PGE2, M-CSF, GM-CSF, MCP-1, and RANKL (Horowitz and Purdon, 1995; Haynes et al., 1997; Rodrigo et al., 2006; Vallés et al., 2008a). These inflammatory mediators lead to further macrophage recruitment, increased osteoclastogenesis, and suppression of osteoblast function (Horowitz et al., 1994; Horowitz and Purdon, 1995; Gallo et al., 2013; Guo et al., 2013). In an indirect co-culture model, reduction of the TNFα level using neutralizing TNFα antibodies or TNFα-siRNA in the conditioned medium from particle-activated murine macrophages (J774/RAW 264.7) resulted in down-regulation of IL-6, PGE2, and GM-CSF in murine osteoblastic cells (MC3T3-E1) (Horowitz and Purdon, 1995; Guo et al., 2013). Similarly, TNFα and IL-1β antibody treatment induced similar effects in a THP-1 and human osteoblast co-culture model under challenge with TiO_2_ rutile and pure Ti wear particles (Vallés et al., 2008a).

Osteoblasts also release soluble mediators and reciprocally modulate the extent of the response initiated by macrophages upon particle stimulation. Much lower levels of TNFα, IL-1β, and NO production were detected in osteoblast-macrophage co-culture models compared with that in macrophages cultured alone (Naskar et al., 2014; Jablonski et al., 2016). Rodrigo et al. (2006) found that, after co-culturing with human osteoblasts, mouse J774 cells reduced TNFα release but enhanced IL-6 secretion, both in the absence or presence of alumina particles (Rodrigo et al., 2006). This anti-inflammatory effect is potentially mediated by PGE2 secreted from osteoblasts, through paracrine action (Horowitz and Gonzales, 1996; Rodrigo et al., 2006). Notably, macrophages can secrete endogenous lipoxin A4 (LXA4) to counteract PMMA-induced cytokine production. Importantly, macrophages could only secrete LXA4 when they were co-cultured with OBs (Li et al., 2009). This indicates that the osteoblast-macrophage interaction also contributes to the resolution of particle-induced inflammation.

### Osteoblast-Osteoclast Interaction

Osteoclasts are the only *in vivo* cells with bone resorption function. They maintain bone metabolism homeostasis by acting synergistically with osteoblasts. The unbalanced crosstalk between them could lead to disruptive bone homeostasis. Osteoblasts interact with osteoclasts *via* the secreted factors RANKL and OPG to maintain a delicate balance in bone remodeling. Binding of RANKL to RANK activates NF-κB signaling pathways that ultimately lead to osteoclastogenesis. OPG is a soluble “decoy receptor” for RANKL and thus a physiologically negative regulator of osteoclastogenesis. The RANKL/OPG expression ratio determines the degree of osteoclast differentiation and function and has been shown to be implicated in the process of osteolysis.

Particle challenge switches mature osteoblastic cells from an anabolic to a more catabolic phenotype (Atkins et al., 2009), leading to increased expression of mediators involved in osteoclastogenesis (TNFα, IL-1β, RANKL, IL-6, PGE2, M-CSF, IL-8, and MCP1) (Dean et al., 1999b; Pioletti and Kottelat, 2004; Atkins et al., 2009; Jonitz-Heincke et al., 2016) and decreased expression of OPG, reduced OPG-to-RANKL ratio (Granchi et al., 2004, 2005; Atkins et al., 2009), and transforming growth factor (TGF-β1) (Dean et al., 1999a; Ma et al., 2010). Conditioned medium from UHMWPE particle-challenged hOBs has been shown to induce large numbers of multinucleated TRAP-positive giant cells from PBMCs at day 7 post-challenge *in vitro* (Granchi et al., 2004, 2005). These studies have also reported that, in contrast, Al_2_O_3_ particles are less active in the induction of osteoclastogenesis (Li and Hastings, 2016), supporting the inert biological behavior of ceramic biomaterials.

Osteoclasts can also secrete extracellular vesicles such as exosomes and microvesicles, to regulate osteoblasts. Recent studies identified a RANKL reverse signaling pathway in osteoclast-osteoblast coupling. Osteoclasts secrete vesicular RANK, which promotes osteogenic bone formation through stimulating osteoblast differentiation (Ikebuchi et al., 2018; Ma et al., 2019). Other signals such as semaphorins (Sema) 4D and neurotrophins also play important roles in communication between osteoclasts and osteoblasts. In addition, the role of non-resorbing osteoclasts in the regulation of osteoblasts under particle challenge might also be interesting to explore as evidence suggests that they are capable of influencing osteoblast function (Kreja et al., 2010; Romeo et al., 2019). However, the role of RANKL reverse signaling, Sema 4D and non-resorbing osteoclasts in the context of aseptic loosening has never been investigated.

In summary, under challenge with wear debris, osteoblasts interact with macrophages and osteoblasts through proinflammatory mediators (Figure 1). These interactions contribute to the recruitment, migration, differentiation, and ultimately activation, of bone-resorbing osteoclasts, thus promoting periprosthetic osteolysis.

## Signaling Pathways in Wear Debris-Activated Osteoblasts

**FIGURE 2:**
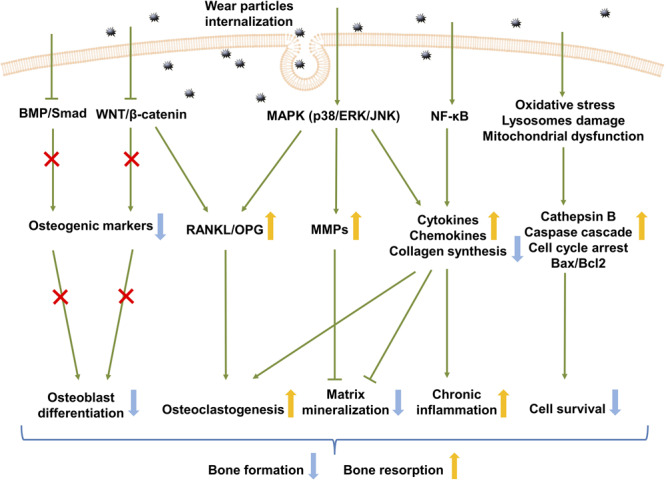
Signaling pathways in wear debris-activated osteoblasts. Wear particle internalization results in activation of several signaling pathways in osteoblasts. The activation of the MAPK and NF-κB signaling pathway up-regulates inflammatory cytokine and chemokine production, which favors chronic inflammation, while down-regulating collagen synthesis. MAPK activation disturbs osteoblastic RANKL/OPG balance and increases MMP production. Osteoclastogenesis is therefore increased while remodeling of the extracellular matrix deposition is inhibited. Wear particles also inhibit the WNT and BMP signaling pathway which mainly contributes to osteoblast differentiation. This ultimately results in decreased bone formation and increased bone resorption. BMP, bone morphogenetic protein; RANKL, receptor activator of nuclear factor kappa-B ligand; OPG, osteoprotegerin; ERK, extracellular signal-regulated kinase; JNK, Jun N-terminal kinase; MMPs, matrix metalloproteinases; NF-κB, nuclear factor-kappa B; Bax, BCL2-associated X protein; Bcl, B cell Leukemia/Lymphoma 2 apoptosis regulator.

Osteoblastic cells serve as an ideal *in vitro* model to study the molecular mechanisms of signaling pathways in the context of wear particles (Figure 2). Elucidating the underlying molecular pathogenesis is considered to be a key in the development of therapeutic strategies for osteolysis.

NF-κB is a master regulator of immune responses, and one of the major signaling pathways activated during aseptic loosening (Lin et al., 2014). It results in the altered production of type I collagen (Vermes et al., 2000; Roebuck et al., 2001), and predominantly activates proinflammatory cytokines (TNFα, IL-1β, and IL-6) and chemokines (IL-8 and MCP1) (Fritz et al., 2002, 2005, 2006). For example, MG-63 cells exposed to Ti particles showed increased binding of the transcription factor NF-κB to the IL-8 promoter, as well as elevated MAPK signaling (ERK, JNK, and p38) activation. Furthermore, IL-8 release was shown to be suppressed by specific inhibitors of the ERK and p38 MAPK pathways, indicating that Ti particle-induced NF-κB-mediated transcriptional activation is MAPK pathway-dependent (Fritz et al., 2005). Another study has shown that the mRNA expression of *CXCR4*, the chemokine receptor for CXCL12, induced by CoCr particles is regulated by the PLC-DAG-PKC pathway, rather than the MAPK/ERK pathway (Drynda et al., 2017), in MG-63 cells.

Glycogen synthase kinase-3 beta (GSK-3β), a member of the β-catenin destruction system, is a central player in canonical WNT signaling. Recently, Geng et al. (2015) showed that Ti-stressed MC3T3-E1 cells exhibited up-regulated GSK-3β expression and suppression of the β-catenin signaling pathway, leading to osteoblastogenesis impairment both *in vitro* and *in vivo* (Geng et al., 2015; Gu et al., 2017). It was shown that lithium chloride (LiCl) (GSK-3β inhibitor) promoted β-catenin nuclear translocation and up-regulated β-catenin signaling activity even in the presence of Ti particles. It also reduced the RANKL/OPG ratio and induced osteoblast differentiation in the presence of Ti particles. Furthermore, blockade of the β-catenin pathway with the ICG-001 inhibitor attenuated the protective effects of LiCl on osteoblast differentiation *in vivo*. These results further implicate the GSK-3β/β-catenin signaling pathway as a mediator of Ti particle-induced osteolysis. A recent study showed that modulation of Wnt/β-catenin and BMP-2 signaling with GSK-3β inhibitor AR28 accelerated bone repair, up-regulated osteoblast differentiation, and attenuated osteoclastogenesis *via* an OPG-associated mechanism in a rat model of instability-induced osteolysis (Amirhosseini et al., 2017).

MAPK is another intracellular signal-transducing molecule that regulates wear debris-induced inflammatory osteolysis. The p38 MAPK signal transduction pathway is activated in Ti particle-challenged MC3T3-E1 cells (Chen et al., 2014; Zhang and Zhang, 2019) as well as in a murine osteolysis model (Chen et al., 2015). Chen et al. (2015) demonstrated that p38 signaling is required for MMP-2 activity in osteoblasts under wear particle-induced conditions. In this study, co-culture of MC3T3 E-1 cells with Ti particles activated the p38 MAPK signaling pathway and triggered MMP-2 expression. Pretreatment with SB203580, a p38 inhibitor, abolished Ti particle-induced *MMP-2* mRNA expression and protein secretion. SB203580 was also reported to inhibit Ti particle-induced inflammatory osteolysis *in vivo* through the down-regulation of RANK/RANKL (Chen et al., 2015) and MMP-9/TNFα (Chen et al., 2012) in murine osteolysis models. Nam et al. (2017) found that the activation of p38 was not observed until 1 h after particle exposure in MC3T3-E1 cells, suggesting that the p38 pathway is activated during the later stages of the response to Ti particles. In their study, ERK and JNK pathways were activated as an early response to Ti particles and found to participate in WNT and BMP signaling pathway suppression and subsequent osteogenesis inhibition. Co-inhibition of ERK and JNK with their specific inhibitors resulted in partial recovery of WNT and BMP signaling activity, as well as ALP activity and collagen synthesis. This result is consistent with the findings of Lee, who showed that ERK1/2–CEBP-β intracellular signaling was activated after Ti particle phagocytosis, resulting in the downstream induction of COX-2 and IL-6 production, but not that of TNFα (Lee et al., 2011; Lee H. G. et al., 2012). Further, inhibiting the MAPK/ERK kinase-1/2 pathway with AZD6244 mitigated inflammatory responses and attenuated inflammatory osteolysis both *in vivo* and *in vitro*. Moreover, ERK1/2 signaling pathway has been shown to mediate Ti particle-induced M-CSF expression in MC3T3-E1 cells (Seo et al., 2007). In addition, CoCrMo metal particles stimulate autophagy in osteoblasts through ERN1-MAPK8, an endoplasmic reticulum (ER) stress signaling pathway, and induce apoptosis.

Internalized wear particles can also exert cytotoxic effects by inducing apoptosis *via* the production of reactive oxygen species (ROS). This leads to not only an increase in lysosome permeability and cathepsin B release, but also mitochondrial dysfunction and DNA damage (Jin et al., 2018). Furthermore, caspase-dependent apoptosis of osteoblasts has been reported. This is attributed to the increase in the expression of proapoptotic proteins (BAX, Caspase-3, and 9) and decrease in that of antiapoptotic proteins (Bcl-2) as well as cellular tumor antigen p53 (Pioletti et al., 2002; Jin et al., 2018; Zhang and Zhang, 2019).

Altogether, these findings suggest that several different intracellular signaling pathways are involved in mediating the adverse effects of wear debris on osteoblasts (Figure 2); however, the number of studies in this context is limited. Therefore, further investigations are required to elucidate the relative importance and complex mechanisms underlying the interactions between these intracellular pathways, as well as the existence of other potential pathways.

## Pharmacological Targeting of Osteoblasts to Limit Periprosthetic Osteolysis: a Summary of *In Vivo* Evidence

Currently, there are no approved medical therapies available for wear particle-induced osteolysis. Increasing evidence shows that osteoblasts serve as an attractive therapeutic target for aseptic loosening. Several studies have used various pharmaceutical compounds targeting osteoblasts both *in vitro* and *in vivo* to enhance osteoblastic function, to modulate inflammatory reactions, and to facilitate bone formation. Thus, in this section, pharmacological targeting of osteoblasts in periprosthetic osteolysis, with particular focus on *in vivo* studies, is summarized (Table 3).

**TABLE 3:** Summary of drug candidates for the treatment of wear particle-associated osteolysis in animal models.

**Drug candidates**	**Signaling pathways in osteoblasts**	**Action**	**Effect of treatment**	**References**
Melatonin, icariin, ghrelin, LiCl, strontium ranelate, teriparatide	Wnt/β-catenin	Activator	Osteoblast proliferation↑, osteoblast differentiation↑, OPG/RANKL↑, osteoclastogenesis↓	Wang et al., 2016; Yu and He, 2016; Gu et al., 2017; Ping et al., 2017; Geng et al., 2018c; Lian et al., 2018; Qu et al., 2019
Statins (e.g., simvastatin)	RhoA/ROCK	Inhibitor	Inflammatory reaction↓, new bone formation↑, osteoclastic bone resorption↓	von Knoch et al., 2005b; Vallés et al., 2013; Zhang et al., 2015
SB203580, triptolide, AZD6244	MAPK (ERK/p38/Jnk)	Inhibitor	Inflammatory cytokine↓, chemokine↓, collagen synthesis↑, autophagy↓, RANK↓, RANKL↓, OPG↑, NF-κB↓	Lee H. G. et al., 2012; Chen et al., 2015; Feng et al., 2018
3-MA	Autophagy	Inhibitor	Osteoblasts apoptosis↓, proliferation↑, osteogenesis↑, inflammatory reaction↓, OPG/RANKL↑, osteoclastogenesis↓	Wang et al., 2015b
Resveratrol, GYY4137	SIRT1-NF-κB	Inhibitor	Osteoblastogenesis↑, inflammatory cytokine↓, chemokine↓, collagen synthesis↑	Liu et al., 2020
Resveratrol, GYY4137	SIRT1-p53	Inhibitor	Osteoblastic apoptosis↓	Deng et al., 2017b

*OPG, osteoprotegerin; RANKL, receptor activator of nuclear factor kappa-*B* ligand; RhoA, ras homolog gene family, member A; ROCK, rho-associated kinase; MAPK, mitogen-activated protein kinase; RANK, receptor activator of nuclear factor-κB; SIRT, sirtuin.*

Ideally, assessing pathogenesis of wear particle-associated osteolysis in clinical cases is the gold standard. Nevertheless, such approaches are challenging because of the necessity of long-term follow up, low occurrence, as well as ethical issues. To date, most studies concerning wear particles have been performed using *in vitro* models. The reported findings have offered insights into cellular functional changes and identified relevant signaling pathways involving the use of high-throughput screening in order to discover potential drugs. However, owing to the involvement of multiple cell lineages that interact with each other, evaluating the effect of wear particles on only one specific cell line or cell signaling pathway among the numerous ones involved in this complex environment is not sufficient for understanding the problem of implant loosening as a whole. Animal models are therefore necessary for further studies. Animal models provide an effective and valid tool for studying the interactions between wear particles and various peri-implant cells. Their strengths include the following: (1) simplicity of experimental procedures and low cost, (2) rapid development of osteolysis, (3) quantitative evaluation of bone loss, and (4) availability of transgenic or knockout models (Langlois and Hamadouche, 2011). However, animal models cannot be used to assess the chronic *in vivo* effects of wear particles because of their short physiological time courses. Importantly, many animal models are non-implant models; therefore, loaded situations (gait and mechanical force) are impossible to evaluate.

Sirtuin 1 (SIRT1) plays an important role in the pathogenesis of aseptic loosening. Metal (TiAl_6_V_4_ and CoCrMo) particles have been shown to down-regulate SIRT1 expression in MC3T3-E1 cells, macrophages, PIO mouse models, and interface membranes from patients with aseptic loosening (Deng et al., 2017a, b). These findings indicate a close link between SIRT1 and aseptic loosening. *In vitro* pharmacological up-regulation and activation of SIRT1 with resveratrol ameliorated the particle-induced osteoblastic apoptosis and inflammatory cytokine expression by suppressing SIRT1-p53 and SIRT1-NF-κB signaling, respectively. Unsurprisingly, *in vivo* SIRT1 activation by resveratrol and GYY4137 protected cells from osteoblast dysfunction, attenuated particle-induced inflammatory responses, and osteolysis in PIO mouse models (Deng et al., 2017b; Liu et al., 2020).

The GSK-3β/Wnt/β-catenin signaling pathway is a mediator of Ti particle-induced osteolysis *in vivo*. Melatonin, icariin, and ghrelin, which activate Wnt/β-catenin signaling, have been shown to rescue Ti particle-impaired cellular function in MSCs and MC3T3-E1 cells *in vitro*, and to attenuate particle-induced osteolysis in animal models (Wang et al., 2016; Ping et al., 2017; Lian et al., 2018; Qu et al., 2019). Furthermore, inhibiting GSK-3β activity with LiCl increased downstream β-catenin expression and mitigated Ti particle-induced suppression of osteogenesis, both *in vitro and in vivo* (Geng et al., 2015; Gu et al., 2017).

Strontium ranelate, which is currently approved for the treatment of post-menopausal osteoporosis, is considered a potential treatment for particle-induced osteolysis (Geng et al., 2018c). Previous studies showed that it can promote osteoblast proliferation, suppress inflammatory osteoclastogenesis both *in vitro* and in wear particle-induced mouse models (Zhu et al., 2016; Karakan et al., 2017; Geng et al., 2018a, b). These effects were dependent on down-regulation of SOST levels to ameliorate subsequent WNT/β-catenin pathway inhibition in osteoblasts, as no protective effect on Ti particle-induced osteolysis was observed in sclerostin^–/–^ mice (Geng et al., 2018c). In addition, a potential role of the BMP signaling pathway should not be neglected (Lee S.-S. et al., 2012; Quade et al., 2020).

Statins, a class of cholesterol-lowering drugs used clinically to reduce the risk of cardiovascular diseases, have been documented to have a beneficial effect on bone metabolism (Zhang et al., 2014). The use of statins is associated with a substantially lower risk of developing femoral osteolysis, and lower revision risk following primary total hip arthroplasty (Thillemann et al., 2010; Lübbeke et al., 2013). Simvastatin pretreatment down-regulated Ti particle-induced IL-6 production in SaOS2 and hOB cells *in vitro* (Vallés et al., 2013). This effect is mediated by inhibition of the HMG-CoA/GGPP/RhoA/ROCK pathway. It also attenuates PMMA particle-induced cytokine response in human monocytes by suppression of the NF-κB signaling pathway (Zhang et al., 2015). Further, this treatment markedly decreased osteolysis and promoted new bone formation in UHMWPE particle-induced osteolysis in a murine calvarial model (von Knoch et al., 2005b, c). Additionally, a protective role for statins in inflammatory reactions has been observed in PBMCs (Wang Z. et al., 2010) and monocytes under wear particle challenge *in vitro* (Laing et al., 2008; Zhang et al., 2015). Moreover, statins suppress osteoclastic bone resorption *in vitro* as well as *in vivo* (Hughes et al., 2007). This evidence strongly suggests that statins are a promising therapeutic agent for the prevention and treatment of aseptic loosening of prostheses.

The MAPK signal transduction pathway, which involves ERK, p38, and JNK, has been reported to play a significant role in wear particle-induced osteolysis (Fritz et al., 2005; Ma et al., 2010). p38/ERK/JNK inhibitors demonstrate a promising therapeutic effect against wear particle-induced osteolysis in PIO animal models by enhancing osteoblast function and suppressing osteoclast formation and function. In detail, the p38 MAPK inhibitor, SB203580, has been reported to have therapeutic effects on wear debris-induced osteolysis both *in vitro* (Chen et al., 2014; Zhang and Zhang, 2019) and *in vivo* (Chen et al., 2012, 2015). The p38 signaling pathway inhibitor, triptolide, has also been shown to inhibit Ti wear particle-induced osteolysis *in vivo* by suppressing osteoclast formation and function, RANKL expression as well as upregulating OPG in osteoblasts (Feng et al., 2018). The ERK pathway is a key inflammatory signaling pathway in wear particle-challenged osteoprogenitor cells (Seo et al., 2007; Lee et al., 2011). AZD6244, a potent ERK pathway inhibitor, attenuated particle-mediated inflammatory osteolysis both *in vivo* and *in vitro* (Lee et al., 2011).

As previously presented, the autophagy pathways play a significant role in peri-implant osteolysis as they can be triggered by wear particles and promote apoptosis as well as inflammatory reaction. Blocking autophagy with 3-MA has been shown to rescue wear particle-induced osteoblast’s apoptosis, functional disturbances *in vitro* (Zhang and Zhang, 2019) as well as osteolysis in animal models (Wang et al., 2015b). Activation of the TWEAK-p38 pathway by hyperoside pretreatment can decrease autophagy and increase cell viability and proliferation, and thus protects MC3T3-E1 cells against Ti particle-induced damage (Zhang and Zhang, 2019).

Anti-resorptive agents such as bisphosphonates, selective estrogen receptor modulators (SERMs), and anabolic drugs that stimulate bone formation, including PTH analogs and sclerostin inhibitors, are current treatments for osteoporosis. Indeed, some of them have been proven to reduce wear particle-induced osteolysis. Teriparatide (PTH 1-34) is also an osteoporosis medication which can increase bone formation *via* PKA (protein kinase A) and Wnt/β-catenin pathways (Tian et al., 2011), and also increases the secretion of OPG. It has been reported to mitigate wear particle-induced osteolysis in a murine calvarial model (Yu and He, 2016). OPG therapy also demonstrated positive effects on wear debris-induced osteoclastogenesis and osteolysis both *in vitro* and *in vivo* (Ulrich-Vinther et al., 2002; von Knoch et al., 2005a). However, the effect of bisphosphonates and OPG primarily inhibits bone resorption through modulating osteoclast function. Their effect on osteoblasts and wear particle-induced osteolysis has not been fully addressed. More studies concerning these drugs are therefore needed.

MSC-based treatment: MSCs possess immunomodulatory abilities, secrete pro-regenerative growth factors and differentiate into osteoblasts and could thus be a promising treatment for particle-associated osteolysis. Local delivery of MSCs or cytokine-primed MSCs to enhance implant integration, to modulate inflammatory reactions and to facilitate bone healing (Lin et al., 2017; Pajarinen et al., 2017), have been proven to be successful both *in vitro* and *in vivo*. However, clinical studies in humans remain to be conducted.

In summary, osteoblasts indeed are promising targets against wear particle-induced periprosthetic osteolysis. Rather than examining the local delivery of osteoblasts, the existing data mainly focus on pharmacologic enhancement of osteoblast function, modulating inflammatory reactions and facilitating bone formation. In addition to those seeking to discover new effective pharmacological reagents, further studies aimed at elucidating the effects of existing drugs on other periprosthetic cell lineages are warranted. Furthermore, improvements in the specificity, safety, and manipulability of these drugs are needed before they can be considered for clinical application.

## Limitations and Future Directions

Osteoblasts may represent a promising therapeutic target for the treatment of aseptic loosening. Indeed, some agents have been indicated to be effective in osteoblast-like *in vitro* cell models (MG-63, SaOS-2, and U-2 OS). However, unlike primary osteoblasts, osteosarcoma cell lines represent only one stage of osteoblastic maturation and phenotype. The reaction of osteoblasts to particles therefore depends on the specific cell lines used, and it seems that osteoblast-like cells only partially reproduce the behavior of primary hOBs under particle stimulation (Saldaña et al., 2011). Scanning electron microscopy has revealed that particulate materials generated from implant wear vary more greatly in shape than commercially produced particles and are more detrimental or inflammatory to osteoblasts. This supports the concept of using primary hOBs and particles retrieved from loosened implants to explore the particle-cell interaction. In addition, across the diversity of cell culture protocols, procedures, reagents, and biomaterials used, osteoblasts show differential responses to various types of wear debris. Further investigations are essential to clarify these discrepancies. For example, if peri-implant cells are differentially influenced by diverse types of particles, then elucidation of the reasons for these differences would be a starting point for material-specific targeting of periprosthetic osteolysis.

Second, to date, communication between peri-implant cell lineages in the context of wear particles as well as their underlying mechanism has not been fully addressed both *in vitro* and *in vivo* and several questions remain. For example: (1) What is the role of RANKL reverse signaling or semaphorins in osteoclast-modulated osteoblastic function change during wear particle challenge? (2) Do osteoblasts communicate with osteoclasts through other pathways rather than OPG/RANKL and inflammatory secretion? (3) What is the involvement of other cell subpopulations (e.g., osteocytes, non-resorbing osteoclasts, OsteoMacs) in the cellular crosstalk during osteolysis and to what extent do they contribute? In addition, the contribution of other factors, such as fluid pressure and mechanical loading, in the progression of wear particle-induced periprosthetic osteolysis are still missing. Further down the road, clinical studies concerning the cytokine profile in patients with aseptic loosening should also be emphasized as it may provide diagnostic tools and predict markers for aseptic loosening. Thus, more studies are needed to fully elucidate these aspects.

Third, as several pathways or molecular mechanisms (e.g., phagocytosis, autophagy, GSK-3β/Wnt/β-catenin, and p38/ERK/JNK) have been identified during the pathology of wear debris-associated osteolysis, further studies are required for the development of drugs targeting these pathways and mechanisms to curtail aseptic loosening. Notably, although some drugs targeting osteoblasts have demonstrated efficacy in cell cultures and even animal models, their translational potential remains to be established. More specifically, many of them are not specific to osteoblasts, which may cause diverse and systematic adverse effects. For example, non-selectively inhibiting the phagocytic pathway, may impair host defenses of macrophages and lead to potential adverse effects such as infection (Gordon, 2016). Additionally, osteoblasts are not the only cells that contribute to the development of aseptic loosening. Thus, pharmacological blockade of one signaling pathway in one cell type alone is unlikely to successfully alleviate the overall effects of wear particles. Thus, a combination of pharmacology targeting on multiple peri-implant cells (not only osteoblasts) as well as their related pathological pathways (intracellular signaling) may be a more feasible strategy to curtail wear particle-induced peri-prosthetic osteolysis in the future. However, improvements in the specificity, safety, and manipulability of these drugs are needed before they can be clinically applied.

## Conclusion

Osteoblasts, which are central actors in bone tissue formation, play a significant role in aseptic loosening. *In vitro* models demonstrate that wear particle exposure to osteoblasts results in the inhibition of multiple cellular functions, release of proinflammatory factors, and activation/inhibition of different signaling pathways. These reactions directly impair osteoblastic bone formation and indirectly contribute to osteoclastic bone resorption. Furthermore, based on these *in vitro* models, effective pharmacological interventions targeting osteoblasts *in vivo* are emerging. Additional studies are needed to delineate the precise roles of osteoblasts in aseptic loosening, and to confirm their potential utility as a therapeutic target.

## Author Contributions

All authors listed have made a substantial, direct and intellectual contribution to the work, and approved it for publication.

## Conflict of Interest

The authors declare that the research was conducted in the absence of any commercial or financial relationships that could be construed as a potential conflict of interest.
